# Osteomuscular symptoms on motorcycles in the city of Rio Branco, Acre, Brazil, West Amazon

**DOI:** 10.1097/MD.0000000000025549

**Published:** 2021-04-23

**Authors:** Narjara Campos de Araújo, Orivaldo Florêncio de Souza, Mauro José de Deus Morais, Francisco Naildo Cardoso Leitão, Italla Maria Pinheiro Bezerra, Luiz Carlos de Abreu, Luciano Miller Reis Rodrigues

**Affiliations:** a ABC Medical School (FMABC) – Santo André – SP; b Multidisciplinary Laboratory of Studies and Scientific Writing in Health Sciences – LAMEECCS, Center for Health Sciences and Sports, Federal University of Acre – UFAC, Rio Branco, AC; c Laboratory of Scientific Writing, Faculty of Medicine of ABC, Santo André, SP; d Public Policy and Local Development Program of the School of Sciences Superior of Santa Casa de Misericórdia de Vitória, Vitória, ES, Brazil; e Cardiorespiratory Research Group, Department of Biological and Medical Sciences, Faculty of Health and Life Sciences, Oxford Brookes University, Headington Campus, Oxford, OX3 0BP, United Kingdom.

**Keywords:** epidemiology, low back pain, musculoskeletal pain, occupational health

## Abstract

Musculoskeletal disorders gradually affect workers in different parts of the world, compromising their occupational health and quality of life. Professionals exposed to these symptoms include the motorcycle taxi driver, whose pain is due to the overuse of the musculoskeletal system and little time to recover it.

To identify the prevalence of musculoskeletal symptoms in motorcycle taxi drivers in the city of Rio Branco, Acre, Brazil, West Amazon.

Cross-sectional study, involving 296 motorcycle taxi drivers in the city of Rio Branco-Acre, Brazil, male, from December 2016 to February 2017. The Nordic Musculoskeletal Questionnaire was used to collect information related to symptoms (pain, discomfort, or numbness) in the last 7 days of work. For the exclusion criteria were, being female; not reside outside the city of Rio Branco, Acre; having less than 3 months of work activity; not be carrying out their work activities at the time of application of the protocol; be limited by clinical or physical issues at the time of application of the protocol. The data obtained in the questionnaire were entered into the Epidata program (Epidata Association, Odense, Denmark) and then transferred to the STATA 10 statistical program (Stata Corp., College Station), for categorization and statistical analysis.

The study population is over 36 years old; most reported having a partner and a higher education level. The average daily working hours of the participants were 12 hours, with the majority working over 12 hours daily. Most of the epidemiological variables factors were associated with musculoskeletal pain when the prevalence and prevalence ratio analyzes were performed. Higher prevalence of musculoskeletal symptoms in the lumbar region is with 17.9%. In the lower limbs, the most affected joint was the ankle (5.7%), followed by the hip (5.07%) and knee (5.07%), respectively. Insomnia was present in 55.35% and self-reported headache in 49.4% of participants.

The musculoskeletal disorders generated by the daily service of motorcycle taxi drivers are directly affecting the quality of life of these professionals.

## 1 Introduction

Musculoskeletal disorders have affected workers in several countries, directly impacting occupational health and decreasing the quality of life of these professionals, which results in a serious problem in the public health area of the worker, consequently leading to incapacity at work and increasing the number of absenteeism in the work sector.^[1,2]^

Several factors are involved in the pain mechanism. Cytokines are fundamental to the inflammatory response and almost all cells secrete as cytokines, which are small proteins and have a role in regulating and influencing the immune response.^[3]^ This release of pro-inflammatory cytokines will lead to the activation and production of immune cells, as well as the release of other cytokines.^[4]^ Several studies with human beings, point out that the IL-6-634-C polymorphism is associated with the appearance of some pathologies as in the case of reduced bone mineral density.^[5]^ In our studies, exams of this nature were not performed to survey this variable.

In 1974, Charles A. Dinarello discovered Interleukins 1α and Interleukin 1β.^[6]^ Studies show the role of these inflammatory cytokines in the regulation of bone homeostasis. Chronic inflammation is usually characterized by an imbalance between bone formation and bone resorption.^[7]^ However, the evidence is limited when it comes to which cytokine is the most important for bone biology. The link between osteoclasts and pro-inflammatory cytokines, especially IL-1, provides an explanation for the association between inflammation and osteoporosis.^[8]^

The tumor necrosis factor alpha is differentiated by its cytotoxic activity for tumor cells through immune cells and, therefore, it was called tumor necrosis factor (TNF).^[7]^ It is expressed as a type II transmembrane protein (mbTNFα), but it can be cut into its soluble form (sTNFα) with increased biological activity.^[9]^ Its functional activity is diverse and stands out for its cellular mediation and pro-inflammatory response by TNFR-I via NF-κB and activating protein (AP)-1^[10]^ and tumor necrosis factor alpha (TNF-α) still acts as a stimulating signaling pathway for cell death through Fas and Caspases.^[9,11]^ TNF-α antagonists, such as etanercept, infliximab, or adalimumab, for treating inflammatory diseases such as in the case of rheumatoid arthritis, proved to be very effective.^[10]^ Similar to IL-6, TNF-α can regulate bone metabolism through the endocrine pathway.^[12]^ However, other advanced studies suggest TNF inhibition as the first option to increase bone density, is not recommended, as conflicting findings regarding the use of bisphosphonate may be important to improve bone density in patients with rheumatoid arthritis, even under strict control conditions.^[13]^

These disturbances end up generating increased economic costs and problems of social order as they are the second largest cause of disability in the world.^[14]^

Among the workers exposed to these types of problems, there is the motorcycle taxi driver, a newly regulated profession in Brazil, that consists of driving a rotating transport that uses a motorcycle, also known as *mototaxi*, to transport people, which, for being a faster and economical means of transportation, adapted easily in cities that needed cheaper transport services and did not have a continuous and structured road network.^[15]^

It is important to highlight that these professionals belong to the category of public transport drivers and the working conditions to which they are subjected daily can be influenced by biomechanical factors, related to the posture adopted during the working day, causing musculoskeletal pain in the lumbar spine with the increase the internal pressure of the intervertebral disc, and the consequent stretching of the ligaments as well as the small joints and nerves.^[16,17]^ Among other factors that may affect the onset of musculoskeletal disorders are psychosocial factors, absence of break during the performance of work activity, sitting position with the back curved forward.^[18]^

These pains result from the overuse of the musculoskeletal system and the short time to recover it.^[19]^ Among the main symptoms, the following stand out: pain, paresthesia, numbness, tingling, decreased sensitivity, and a feeling of heaviness or fatigue affecting mainly the upper limbs.^[20]^ Musculoskeletal symptoms are directly related to body movement.^[21]^ Studies carried out in several countries have confirmed the increase in musculoskeletal diseases in several working classes,^[22]^ resulting in a high prevalence in the workplace and being considered a relevant problem for workers’ health.^[23]^

Other factors contribute to the onset of these pains, such as traffic stress, conflicts with car drivers, and too much exposure to the sun,^[24]^ in addition to daily exposure to motorcycle vibration, repeated manual activities, asymmetric and static posture when driving the motorcycle for several hours a day they can cause the appearance of musculoskeletal symptoms in various parts of the body.^[25]^

It is important to highlight that, on the scale of painful disorders that affect men, low back pain is a major cause of morbidity and disability, second only to headache.^[26,27]^

Little is known about the health conditions and illnesses that affect motorcycle taxi drivers and their relationship with the work developed. Therefore, there is the need to know and study the health reality of these professionals. Thus, this study aimed to identify the prevalence and associated factors of musculoskeletal symptoms, in the last 7 days among these professionals.

## 2 Methods

### 2.1 Study design

This is a cross-sectional population-based study^[28]^ carried out with 296 motorcycle taxi drivers from the city of Rio Branco, in Acre.

### 2.2 Study location and period

The research was developed in the city of Rio Branco, Acre, Brazil, from December 2016 to February 2017.

### 2.3 Study population and eligibility criteria

In determining the minimum sample size, the expected prevalence of 50% was adopted, with the precision set for a sampling error of 0.06 and a 95% confidence level. To protect against the effects of no response, being considered at 12%, the final sample size was estimated at 296 motorcycle taxi drivers.

The inclusion criterion in the research was to be working as a motorcycle taxi driver at the time of the research, duly registered with Motorcycle Taxi Drivers Union of Rio Branco, Acre – SINDMOTO. The participants agreed to participate the survey and had no mental problems to better understand the questions.

For the exclusion criteria were being female; not reside outside the city of Rio Branco, Acre, Brasil; having less than 3 months of work activity; not be carrying out their work activities at the time of application of the protocol; be limited by clinical or physical issues at the time of application of the protocol.

### 2.4 Data collection and instrument

A structured questionnaire was used with questions grouped in blocks containing self-reported morbidities. The demographic and socioeconomic variables considered included age, marital status, education. For data analysis, the age group variable was stratified into: less than or equal to 35 years and over 35 years. In the socio-family aspects, the education variable was categorized by years of study: 4 years or less, between 5 and 8 years, and above 9 years of study. The marital status was dichotomized into: with a partner and without a partner.

This instrument, called Nordic Musculoskeletal Questionnaire – NMQ, was used to collect information related to the symptoms (pain, discomfort, or numbness) related to the last 7 days of activity since the application of the said protocol. The following parts were evaluated: cervical/neck, shoulders, thoracic/dorsal, elbows, wrist/hands/fingers, lumbar, hip/thighs, knees, and ankles/feet. This questionnaire was developed by Kuorinka et al^[29]^ and validated by Dickinson et al^[30]^ for the analysis of musculoskeletal symptoms most frequently found in an occupational environment. The translation and validation of the questionnaire into the Portuguese language were carried out by Pinheiro et al.^[31]^ The following question was used to assess each part of the body:

a) considering the last 7 days, have you had any problems (such as pain, discomfort, and numbness)?

We applied a closed semi-structured protocol to assess questions about self-report and the characteristics that involved years of study, occupation time as motorcycle taxi drivers, daily working hours, self-report insomnia, self-report headache, discomfort of driving, excessive working hours. These questions were intended to assess a possible correlation of these factors with the onset of musculoskeletal pain.

### 2.5 Data analysis

The analysis of factors associated with musculoskeletal pain in the last 7 days took place in 2 stages. Initially, the independent variables that showed associations with musculoskeletal pain in the last 7 days at a value of *P* < .20 (chi-square test for heterogeneity) were candidates to compose the multiple model. Subsequently, factors associated with musculoskeletal symptoms were identified by means of multiple logistic regression, using the backward elimination procedure to select the variables of the multiple model. Variables with *P* < .05 were considered as factors associated with musculoskeletal pain.

The data collected through the questionnaire were entered into the Epidata program (Epidata Association, Odense, Denmark) and then transported to the STATA 10 statistical program (Stata Corp., College Station), for categorization and statistical analysis.

### 2.6 Ethical and legal aspects of the research

To conduct the research, the ethical aspects of research involving human beings were respected and only started after the assent of the ethics committee by the Research Ethics Committee of the Federal.

The project was approved by the Research Ethics Committee of the Federal University of Acre, Protocol number 28713114.9.0000.5010. Opinion number: 574.477. Reporting date: 03/27/2014.

## 3 Results

**Figure 1:**
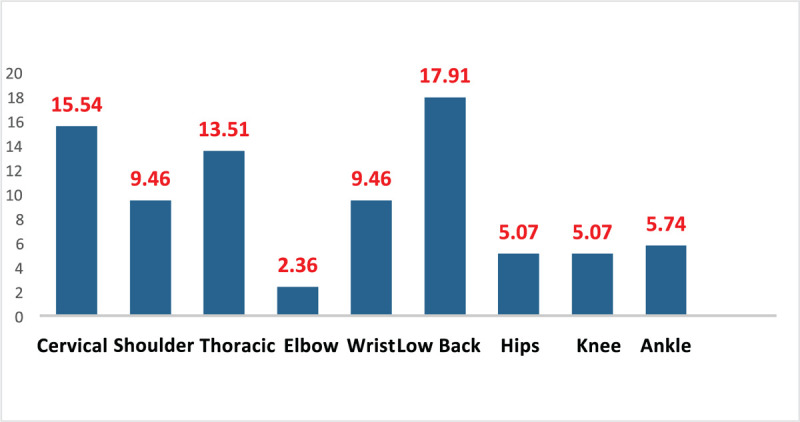
The regions of the body and the prevalences found, in the sequences that emerged in the data collection instrument. Graph 1: Prevalence of musculoskeletal pain by body region in the last 7 d in motorcycle taxi drivers from Rio Branco, Acre, 2017 (*Source*: The author's own).

Considering the period of the last 7 days (Fig. 1), the study found a higher prevalence of musculoskeletal symptoms in the lumbar region, with 17.9%. Then, the highest frequency of symptoms was reported in the cervical (15.5%) and thoracic (13.5%) regions. In the upper limbs, symptoms were reported in the shoulders (9.4%), followed by the wrists (9.4%) and in the lower limbs, the most affected joint was the ankles (5.7%), followed by the hip (5.07%) and knees (5.07%), respectively.

The prevalence of musculoskeletal symptoms was reported by taxi drivers from southwest Nigeria, being cervical (67%), right and left wrists (18%, 20%), upper back (29%), middle back, lower the back (29%), the lower part (30%), and the buttock (19%).^[32]^

Table 1 shows the prevalence of musculoskeletal pain in the last 7 days, distributed among the variables sociodemographic aspects, occupational activity, and stress factors in traffic. The study population is over 36 years old (42.8%), and the majority reported having a partner (43.2%) and a higher education level (46.0%). The average daily working hours of the participants were 12 hours, with the majority (58.6%) working over 12 hours daily and 51.7% reported having between 5 and 10 years in the profession. The total of 58.6% worked more than 12 hours a day and those who took a break to eat for more than an hour a day represented 42.9% of the participants. Insomnia was present in 55.35% and self-reported headache in 49.4% of participants.

**Table 1:** Prevalence and prevalence ratio of musculoskeletal pain in the last 7 d by age, marital status, schooling, and occupational aspects of motorcycle taxi drivers in Rio Branco, 2017.

Variables	n	%	OR	(CI: 95%)	*P*
Age					
35 yr or less	93	41.9	1		
36 yr or more	203	42.8	1.03	(0.63, 1.70)	.82
Marital status					
Without partner	74	40.5	1		
With partner	222	43.2	1.11	(0.65, 1.90)	.84
Years of study					
9 yr or more	176	46.0	1		
Between 5 yr and up to 8 yr	74	41.8	0.84	(0.48, 1.46)	.49
4 yr or less	46	30.4	0.51	(0.25, 1.02)	.60
Time of occupation as mototaxi					
Up to 5 yr	129	34.8	1		
Between 5 and 10 yr	56	51.7	2.00	(1.06, 3.79)	.32
10 yr or more	111	46.8	1.64	(0.97, 2.76)	.60
Daily work day					
Less than 12 h	266	40.6	1		
12 h or more	29	58.6	2.07	(0.95, 4.51)	.67
Break time for meals					
60 min or more	261	42.9	1		
Less than 60 min	35	40.0	0.88	(0.43, 1.82)	.44
Less than 60 min					
No	231	38.9	1		
Yes	65	55.3	1.94	(1.11, 3.39)	.19
Self-report of headache					
No	110	30.9	1		
Yes	186	49.4	2.18	(1.33, 3.59)	.02

The explanatory variables selected as candidates to compose the final model were: years of study, occupation time as motorcycle taxi drivers, daily working hours, self-report of insomnia, self-report of headache, and discomfort when driving.

When performing an analysis with the confidence index between the variables, Table 1, participants over the age of 36 years showed a 1.03 greater chance of developing musculoskeletal symptoms compared to those under the age of 36 years. Those who claimed to have a partner showed a 1.11 greater chance of being affected by musculoskeletal symptoms compared to those who were single.

There was a tendency to feel more musculoskeletal pain among those who had 9 or more years of study when compared to participants with 4 years or less (*odds ratio* [OR]: 0.51; 95% CI: 0.25, 1.02). Less study has become a protective factor against musculoskeletal symptoms. Participants with study time between 5 and 10 years were more likely to have symptoms (OR: 2.00; 95% CI: 1.06, 3.79) in contrast to those with higher education. Individuals who claimed to have been in the service for between 5 and 10 years showed 2.00 times greater chances of being affected by musculoskeletal symptoms when compared to those with up to 5 years in the profession. Having up to 5 years in the profession was a protective factor for musculoskeletal symptoms. When compared with participants who have had an occupation time of more than 10 years, those continue to have an even greater chance of developing musculoskeletal pain compared to those.

In the same Table 1, participants with a workday greater than 12 hours a day were more likely to have musculoskeletal symptoms (OR: 2.07; 95% CI: 0.95, 4.51) compared to those with shorter hours. The pause of less than an hour for less food characterized a chance of developing musculoskeletal symptoms in these professionals. Self-reported insomnia was 1.94 times more likely to develop musculoskeletal pain when compared to those who were not affected by it. The self-reported headache by the participants showed a greater chance of presenting musculoskeletal pain (OR: 2.18; CI: 95%, 1.33, 3.59) in the last 7 days in relation to those who did not manifest it.

When the prevalence and odds ratio of musculoskeletal pain in the last 7 days due to stressful traffic factors were evaluated (Table 2), 42.5% manifested musculoskeletal pain in the last 7 days (OR: 1.00; 95% CI: 0.5, 1.68). Among those who had conflicts with drivers, 45.7% showed musculoskeletal symptoms in the period (OR: 1.17; 95% CI: 0.66, 2.08). Having conflicts with passengers was more likely to develop musculoskeletal symptoms than those who did not.

**Table 2:** Prevalence and odds ratio of musculoskeletal pain in the last 7 d due to stress factors in the traffic of the motorcycle taxi drivers of Rio Branco-AC, 2017.

Variables	n	%	OR	(CI 95%)	*P*
Traffic Jam					
No	80	42.5	1		
Yes	216	42.5	1.00	(0.59, 1.68)	.89
Conflicts with car drivers					
No	237	41.7	1		
Yes	59	45.7	1.17	(0.66, 2.08)	.79
Conflicts with passengers					
No	255	42.3	1		
Yes	41	43.9	1.06	(0.54, 2.07)	.52
Conflicts with service or co-workers					
No	236	39.4	1		
Yes	60	55.0	1.87	(1.06, 3.32)	.31
Motorcycle maintenance					
No	230	40.0	1		
Yes	66	51.2	1.59	(0.91, 2.76)	.97
Discomfort while driving					
No	242	35.5	1		
Yes	54	74.0	5.18	(2.67, 10.0)	≤.01
Excessive working hours					
No	191	34.5	1		
Yes	105	57.1	2.52	(1.54, 4.11)	≤.01
Fear of violence					
No	107	30.8	1		
Yes	189	49.2	2.17	(1.31, 3.58)	.02

Regarding the variable conflicts with service colleagues or union, 55% (OR: 1.87; 95% CI: 1.06, 3.32) were more likely to have musculoskeletal symptoms than those who did not. Regarding the maintenance of the motorcycle, it was demonstrated that the motorcycle taxi drivers responsible for the service had 1.59 (95% CI: 0.91, 2.76) times more likely to develop musculoskeletal symptoms in contrast to those who did not use it.

The prevalence magnitude was 74% (95% CI: 2.67, 10.0) for discomfort when driving the motorcycle. This variable proved to be highly stressful. Professionals who practiced an extensive workday were given 2.52 (95% CI: 1.54, 4.11) times more likely to present musculoskeletal symptoms compared to those who did not have it. In the data analysis, individuals showed 2.17 greater chance (95% CI: 1.31, 3.58) of having musculoskeletal symptoms when the variable fear of violence was analyzed (Table 2).

Table 3 shows the factors associated with musculoskeletal pain in the last 7 days reported by the research participants. Participants with study time between 5 and 8 years were more likely to develop musculoskeletal symptoms compared to those aged 4 years or less. Self-reported insomnia was 1.88 times more likely to develop pain in the last 7 days, showing statistically significant associations in relation to those who did not report headaches. Regarding discomfort while driving, it was found 4.70 times more likely to develop musculoskeletal symptoms in those who reported it, compared to those who did not show such discomfort.

**Table 3:** Factors associated with musculoskeletal pain in the last 7 d in motorcycle taxi drivers of Rio Branco – AC, 2017.

Variables	OR	(CI 95%)	*P*
Years of study			
9 yr or more	1		
Between 5 yr and 8 yr	0.92	(0.49, 1.71)	.10
4 yr or less	0.40	(0.18, 0.89)	.26
Time working as a motorcycle taxi driver			
Until 5 yr	1		
Between 5 and 10 yr	2.67	(1.29, 5.52)	.08
10 yr or more	2.16	(1.18, 3.97)	.12
Daily working hours			
Less than 12 yr	1		
12 h or more	2.57	(1.08, 6.09)	.32
Self-report of insomnia			
No	1		
Yes	1.88	(1.00, 3.55)	.50
Self-report of headache			
No	1		
Yes	1.79	(1.03, 3.12)	.39
Discomfort while driving			
No	1		
Yes	4.70	(2.28, 9.70)	≤.01
Excessive working hours			
No	1		
Yes	2.44	(1.40, 4.23)	≤.01

## 4 Discussion

There was a higher prevalence of musculoskeletal symptoms in the lumbar region, with 17.9%. The study population is over 36 years old, most reported having a partner and a higher education level. The average daily working hours of the participants were 12 hours, with the majority working over 12 hours daily. Insomnia was highlighted, along with a high degree of headache in the self-report assessment. Most of the epidemiological variables and variables related to stress factors were associated with musculoskeletal pain when the prevalence and prevalence ratio analyzes were performed.

These postural stress conditions combined with muscular effort are factors that can affect low back pain.^[33]^ In a study conducted in Tehran with carriers of the Grand Bazaar, it was found that the prevalence of musculoskeletal disorders in the last 7 days in these workers was 23.9%.^[34]^

In a study carried out with taxi drivers from a company in the city of Fukuoka in Japan, the prevalence of 45.8% of low back pain^[35]^ was found in these professionals. In a survey conducted with hairdressers in Ethiopia, it was found that the incidence of low back pain was 50.5% of the individuals interviewed.^[36]^

In the city of Taipei, 51% of the 1242 participating taxi drivers experienced low back pain.^[37]^ In Brazil, a cross-sectional study identified a 28% prevalence of lumbar spine pain in truck drivers.^[38]^ Likewise, in the city of Rio Branco in Acre, Brazil, a study involving professional taxi drivers in the city, observed a prevalence of 49.5% of low back pain^[39]^ among the participants. Such results corroborate with the findings of this research showing that workers who exercise the profession most of the time in a sitting position, tend to develop musculoskeletal symptoms.

Neck pain is among 1 of the 4 most commonly reported musculoskeletal disorders.^[40,41]^ Although this symptom is not associated with high morbidity, the high prevalence and episodic nature of neck pain incurs substantial costs,^[42]^ results similar to the present study, even though it is not directly identified that these pains are related to the profession of motorcycle taxi drivers, the great hours worked daily may lead to believe that there is a correlation.

A large daily workload was also identified, reaching 58.6% of respondents, with a high prevalence factor of OR of 2.07 with musculoskeletal pain, results similar to a study carried out with bus drivers in the city of Hong Kong^[43]^ and with motoboys in the city of Porto Alegre, Rio Grande do Sul (RS), Brazil.^[44]^

There was also a high response rate (18.92%) among the participants of this research, when asked about the musculoskeletal pain referred in the upper limbs region, adding the region of shoulders and wrists. When seeking to characterize the work and health conditions of urban public transpotation drivers in the city of Florianopólis, Santa Catarina, similar results were found,^[45]^ as well as is the city of Jequié, Bahia (BA), Brazil.^[46]^

A study carried out in 2003 with drivers in the cities of São Paulo and Belo Horizonte, in the southeast of Brazil, revealed that 37.4% and 36.3%, of these professionals, respectively, attended at most the 4th grade of elementary school.^[47]^ Low education is a predominant factor among motorcycle taxi drivers, as demonstrated by a survey conducted with these professionals in the city of Paraíba, PB, Brazil.^[48]^ Contradictorily in a study conducted with bankers from Ethiopia, it was evidenced that the level of education was also significantly associated with the development of musculoskeletal disorders, those with low education were 4.2 times more likely to develop musculoskeletal disorders than those who had a master's title.^[49]^ In new studies, the level of education was presented in a high way with 46% of those surveyed, presenting a longer study time.

It was inferred that more years of schooling protect motorcycle taxi drivers from musculoskeletal symptoms. This factor could possibly be explained by the fact that the longer the study period, the greater the chances of motorcycle taxi drivers having knowledge about health and quality of life.

The motorcycle taxi driver needs to be aware that his driving ability is limited in the early morning and/or after sleep deprivation, which can increase the risk of falls and being involved in accidents.^[50]^ The insomnia variable showed a high prevalence with musculoskeletal pain with a OR 1.94 with 55.3% of motorcycle taxi drivers, raising a great concern in this regard.

In this study, it was inferred that headaches in motorcycle taxi drivers have a strong association with musculoskeletal symptoms, although it was not verified whether they already had this condition before they became motorcycle taxi drivers.

The precarious conditions, discomfort when riding the motorcycle, fear of violence, work stress, and prolonged exposure to the sun can contribute to manifestations of varying degrees of headaches in motorcycle taxi drivers.^[51]^ In research aiming to assess risk factors and correlate with pain, it was shown that chronic headache is 4 times greater in the group of individuals with musculoskeletal symptoms than in those without.^[52]^

It points out to the need for actions focusing on public health policies^[53]^ that contributes to promoting the quality of life of this population, in the sense of collaborating with the promotion of occupational health. It is known that worker's health is a topic that needs to be widely discussed and that the assistance provided to this population must focus from prevention and promotion actions to actions aimed at the control and treatment of health problems that affect them. It also emphazised the importance of research that can contribute to new paths in obtaining public health in a given community, as well as knowledge transfer.^[54]^

Thus, during the long working day, motorcycle taxi drivers tend to adopt different postures, which leads to the appearance of these musculoskeletal symptoms, as there is excessive tension in each muscle group resulting in the appearance of pain in various parts of the body.

On the other hand, we observe that these professionals carry out this long workday for financial reasons, with the need for individual and family survival, which means that they do not worry about performing preventive treatments in relation to posture, which will generate consequently, these health problems.

### 4.1 Study limitation

We know that analyses such as high-resolution ultrasound, magnetic resonance, X-ray, among others, are efficient and reliable mechanisms in the clinical area to identify various health problems, including musculoskeletal problems.

The ultrasound exam is able to outline the muscle fibers detecting muscle traumas and neuromuscular diseases^[55]^ and provides a qualitative and/or quantitative assessment of tendon elasticity promoting the early diagnosis of tendinopathy.^[56]^

Even knowing the quality of these exams, our research was focused on the application of 2 protocols, 1 on the participants’ socio-epidemiological data and the second NMQ, assessment protocol, that it is already validated in several countries including Brazil.

The evaluation process of this protocol is based on several questions related to the health of this public, where we are depending exclusively on the individual and personal responses of each one, so we could not use clinical evaluations.

## 5 Conclusion

The excess of hours worked proves to be a high risk factor for the development of musculoskeletal symptoms in this professional category.

## Acknowledgments

We thank the ABC Medical School for providing all the support necessary for this review. We would also like to thank all the motorcycle taxi drivers who participated in this research, contributing to discoveries relevant to the health conditions of this class. To the Secretaria de Estado de Saúde do Acre (SESACRE), the Federal University of Acre (UFAC) and the Centro Universitário Saúde FMABC, for the interinstitutional partnership through agreement n°. 007/2015, for the training of health professionals in Acre, Western Amazon, Brazil.

## Author contributions

Narjara Campos de Araújo: data collection, experiments, and writing the manuscript.

Orivaldo Florêncio de Souza: data collection, carrying out experiments, and writing the manuscript. Write the manuscript, follow the journal's guidelines, and review the statistical analysis.

Mauro José de Deus Morais: supervised the study, wrote the manuscript, wrote the introduction and discussion section, and gave final approval to the version submitted for publication.

Francisco Naildo Cardoso Leitão: supervised the study, wrote the manuscript, wrote the introduction and discussion section, and gave final approval to the version submitted for publication.

Italla Maria Pinheiro Bezerra: supervised the study, wrote the manuscript, wrote the introduction and discussion section, and gave the final approval of the version submitted for publication.

Luiz Carlos de Abreu: supervised the study, wrote the manuscript, wrote the introduction and the discussion section, and gave the final approval of the version submitted for publication.

Luciano Miller Reis Rodrigues: oriented the study, wrote the manuscript, wrote the introduction and the discussion section, and gave the final approval of the version submitted for publication.

**Conceptualization:** Mauro José De Deus Morais, Narjara Campos de Araújo, Orivaldo Florêncio de Souza, Francisco Naildo Cardoso Leitão, Italla Maria Pinheiro Bezerra, Luiz Carlos de Abreu, Luciano Miller Reis Rodrigues.

**Formal analysis:** Orivaldo Florêncio de Souza.

**Methodology:** Mauro José De Deus Morais, Orivaldo Florêncio de Souza.

**Supervision:** Italla Maria Pinheiro Bezerra.

**Validation:** Italla Maria Pinheiro Bezerra, Luciano Miller Reis Rodrigues.

**Visualization:** Mauro José De Deus Morais, Luciano Miller Reis Rodrigues.
